# Supplementary material for: HIVprotI: an integrated web based platform for prediction and design of HIV proteins inhibitors
Source: J Cheminform. 2018 Mar 9;10:12. doi: 10.1186/s13321-018-0266-y (PMC5845081; doi:10.1186/s13321-018-0266-y)
Supplement: Supplementary file 2 — Additional file 2. Source code of HIVProtI web server. [file 13321_2018_266_MOESM2_ESM.zip › HIVprotI_Source-code/hivprotI/weka-3-6-6/doc/weka/experiment/TaskStatusInfo.html]

TaskStatusInfo


---


|  |  |  |
| --- | --- | --- |
| **Overview** \| **Package** \| **Class** \| **Tree** \| **Deprecated** \| **Index** \| **Help** \| **Weka's home** | |  |
| **PREV CLASS**   **NEXT CLASS** | **FRAMES**    **NO FRAMES**     **All Classes** |  |
| SUMMARY: NESTED \| FIELD \| CONSTR \| METHOD | DETAIL: FIELD \| CONSTR \| METHOD |  |


---


## weka.experiment Class TaskStatusInfo

```
java.lang.Object
  weka.experiment.TaskStatusInfo
```

**All Implemented Interfaces:**: java.io.Serializable, RevisionHandler

---

``` public class TaskStatusInfo extends java.lang.Object implements java.io.Serializable, RevisionHandler ```

A class holding information for tasks being executed
on RemoteEngines. Also holds an object encapsulating any returnable result
produced by the task (Note: result object must be serializable). Task
objects execute methods return instances of this class. RemoteEngines also
use this class for storing progress information for tasks that they
execute.

**Version:**
:   $Revision: 1.5 $

**Author:**
:   Mark Hall

**See Also:**: Serialized Form

---

| **Field Summary** | |
| --- | --- |
| `static int` | `FAILED` |
| `static int` | `FINISHED` |
| `static int` | `PROCESSING` |
| `static int` | `TO_BE_RUN` |


**Constructor Summary**

`TaskStatusInfo()`


| **Method Summary** | |
| --- | --- |
| `int` | `getExecutionStatus()`             Get the execution status of this Task. |
| `java.lang.String` | `getRevision()`             Returns the revision string. |
| `java.lang.String` | `getStatusMessage()`             Get the status message. |
| `java.lang.Object` | `getTaskResult()`             Get the returnable result of this task. |
| `void` | `setExecutionStatus(int newStatus)`             Set the execution status of this Task. |
| `void` | `setStatusMessage(java.lang.String newMessage)`             Set the status message. |
| `void` | `setTaskResult(java.lang.Object taskResult)`             Set the returnable result for this task.. |

**Methods inherited from class java.lang.Object**

`equals, getClass, hashCode, notify, notifyAll, toString, wait, wait, wait`

**Field Detail**

### TO\_BE\_RUN

```
public static final int TO_BE_RUN
```

**See Also:**: Constant Field Values

---


### PROCESSING

```
public static final int PROCESSING
```

**See Also:**: Constant Field Values

---


### FAILED

```
public static final int FAILED
```

**See Also:**: Constant Field Values

---


### FINISHED

```
public static final int FINISHED
```

**See Also:**: Constant Field Values


**Constructor Detail**

### TaskStatusInfo

```
public TaskStatusInfo()
```


**Method Detail**

### setExecutionStatus

```
public void setExecutionStatus(int newStatus)
```

:   Set the execution status of this Task.

    :   **Parameters:**: `newStatus` - the new execution status code

---


### getExecutionStatus

```
public int getExecutionStatus()
```

:   Get the execution status of this Task.

    :   **Returns:**: the execution status

---


### setStatusMessage

```
public void setStatusMessage(java.lang.String newMessage)
```

:   Set the status message.

    :   **Parameters:**: `newMessage` - the new status message

---


### getStatusMessage

```
public java.lang.String getStatusMessage()
```

:   Get the status message.

    :   **Returns:**: the status message

---


### setTaskResult

```
public void setTaskResult(java.lang.Object taskResult)
```

:   Set the returnable result for this task..

    :   **Parameters:**: `taskResult` - the new returnable result for the task. null if no result is returnable.

---


### getTaskResult

```
public java.lang.Object getTaskResult()
```

:   Get the returnable result of this task.

    :   **Returns:**: an object encapsulating the result of executing the task. May be null if the task has no returnable result (eg. a remote experiment task that sends its results to a data base).

---


### getRevision

```
public java.lang.String getRevision()
```

:   Returns the revision string.

    :   **Specified by:**: `getRevision` in interface `RevisionHandler`
    :   **Returns:**: the revision


---


|  |  |  |
| --- | --- | --- |
| **Overview** \| **Package** \| **Class** \| **Tree** \| **Deprecated** \| **Index** \| **Help** \| **Weka's home** | |  |
| **PREV CLASS**   **NEXT CLASS** | **FRAMES**    **NO FRAMES**     **All Classes** |  |
| SUMMARY: NESTED \| FIELD \| CONSTR \| METHOD | DETAIL: FIELD \| CONSTR \| METHOD |  |


---
